# Root volume measurements of maxillary canines and lateral incisors in patients with unilateral maxillary canine impaction

**DOI:** 10.1590/2177-6709.29.4.e242416.oar

**Published:** 2024-09-02

**Authors:** Mostafa SHAHABI, Hossein Hosseini ZARCH, Zahra SHADMAN, Farzaneh AHRARI

**Affiliations:** 1 Department of Orthodontics, School of Dentistry, Mashhad University of Medical Sciences (Mashhad, Iran).; 2 Oral and Maxillofacial Diseases Research Center, School of Dentistry, Mashhad University of Medical Sciences (Mashhad, Iran).; 3 Student Research Committee, School of Dentistry, Mashhad University of Medical Sciences (Mashhad, Iran).; 4 Dental Research Center, School of Dentistry, Mashhad University of Medical Sciences (Mashhad, Iran).

**Keywords:** Cone-beam computed tomography, Impacted tooth, Incisor, Tooth root, Buccal canine impaction, Tomografia computadorizada de feixe cônico, Dente impactado, Incisivo, Raiz do dente, Impacção de canino por vestibular

## Abstract

**Objective:**

This study aimed to assess root volumes of maxillary canines and adjacent lateral incisors in patients with unilateral maxillary canine impaction.

**Methods:**

This cross-sectional study was performed on cone-beam computed tomography (CBCT) scans of 100 patients (49 females and 51 males) with unilateral maxillary canine impaction. The images were loaded in Planmeca Romexis Viewer, and root layers between the cementoenamel junction and apex were reconstructed at 600-µm intervals. At each layer, the root boundary was marked, and finally, the root volume was calculated by multiplying the layers’ area by the thickness of 600 µm. The root size of canines and lateral incisors was compared between the impaction and normal eruption sides.

**Results:**

Sixty-two patients showed buccal canine impaction, and 38 presented palatal impaction. The mean root volume of canines on the impaction side was significantly greater than that on the normal eruption side; either the tooth was buccally or palatally impacted (*p*<0.001). The lateral incisors on the side of buccally-impacted canines showed a significantly smaller root volume than that of the contralateral side (*p*<0.001). However, there was no significant difference in the root size of lateral incisors between the two sides in cases presenting palatal canine impaction (*p*=0.177).

**Conclusion:**

The difference in root volume of canines between the two sides can serve as an indicator of canine impaction. The reduction in the root size of the lateral incisor on the side of the buccally impacted canine may be due to root resorption created by pressure from the canine’s crown.

## RESUMO

**Objetivo:**

Este estudo teve como objetivo avaliar o volume radicular de caninos e incisivos laterais superiores em pacientes com impacção unilateral de caninos superiores.

**Métodos:**

Esse estudo transversal foi realizado em tomografias computadorizadas de feixe cônico (TCFC) de 100 pacientes (49 mulheres e 51 homens) com impacção unilateral de caninos superiores. As imagens foram abertas no Planmeca Romexis Viewer e a raiz foi reconstruída em camadas desde a junção amelocementária até o ápice radicular, em intervalos de 600 µm. Em cada camada foi marcado o limite da raiz e, então, o volume da raiz foi calculado multiplicando-se a área das camadas pela espessura de 600 µm. O tamanho da raiz dos caninos e incisivos laterais foi comparado entre os lados com impacção e sem impacção.

**Resultados:**

Sessenta e dois pacientes apresentaram impacção de caninos por vestibular e 38 apresentaram impacção por palatino. O volume médio da raiz dos caninos no lado da impacção foi significativamente maior do que no lado sem impacção; independentemente de o dente estar impactado por vestibular ou palatino (*p*<0,001). Os incisivos laterais vizinhos dos caninos impactados por vestibular apresentaram volume radicular significativamente menor do que os do lado contralateral (*p*<0,001). No entanto, não houve diferença significativa no tamanho da raiz dos incisivos laterais entre os dois lados, nos casos com impacção dos caninos por palatino (*p*=0,177).

**Conclusão:**

A diferença no volume da raiz dos caninos entre os dois lados pode servir como um indicador da impacção do canino. A redução no tamanho da raiz do incisivo lateral vizinho ao canino impactado por vestibular pode ser causada pela reabsorção radicular criada pela pressão da coroa do canino.

## INTRODUCTION

Maxillary canines are the most susceptible teeth to impaction, after the third molars. The impaction appears in approximately 1% to 5% of the population, predominantly among females.^1^ ^-^ ^3^ Maxillary canine impaction generally manifests unilaterally and is more often detected on the palatal rather than the buccal side of the dental arch.^2^ ^-^ ^4^ It is hypothesized that the etiology of buccal canine impaction lies in the lack of adequate space in the dental arch. Conversely, the palatal displacement of canines may be attributable to genetic factors or linked with the agenesis or malformation of lateral incisors.^3^ ^,^ ^5^ Furthermore, the prolonged retention of primary canines, ankylosis or cystic lesions in permanent canines, and the presence of a cleft palate can sporadically result in canine impaction.

Maxillary canines play a great role in facial and dental esthetics and masticatory function. An early diagnosis of canine displacement can provide clinicians with the opportunity for timely intervention and prevention of sequelae related to tooth impaction, such as malocclusion or root resorption.^6^ ^-^ ^9^ Radiography has been widely employed to discern anatomical structures and the positioning of unerupted teeth. Although various techniques -such as periapical, occlusal, panoramic, lateral, or posteroanterior cephalometry- have been utilized for the prediction and localization of impacted canines,^10^ ^-^ ^12^ the advent of 3D imaging has offered remarkable opportunities that were not achievable with previous methods. This technology allows for the analysis of images in any dimension and provides more precise and reproducible measurements of teeth and adjacent structures.^13^ ^-^ ^17^

The volume of the root in maxillary canines and lateral incisors could serve as a predictive or etiological factor for canine impaction. Nevertheless, there is little information concerning the root size of impacted canines and adjacent teeth in patients experiencing buccal as opposed to palatal canine impaction. Therefore, the present study aimed to establish root volume measurements of maxillary canines and adjacent lateral incisors utilizing 3D reconstructed cone-beam computed tomography (CBCT) images in patients affected by unilateral maxillary canine impaction. 

## MATERIAL AND METHODS

### STUDY DESIGN

This retrospective cross-sectional study was executed on CBCT scans of 100 patients (51 males, 49 females), who were older than 16 years and manifested unilateral maxillary canine impaction on either the left or right side of the maxilla. The images were originally taken for orthodontic treatment purposes at a private Oral and Maxillofacial Radiology Center. Patients with systemic diseases influencing tooth eruption, those with craniofacial syndromes such as cleft lip and palate, and subjects exhibiting malformed or missing lateral incisors were excluded from the analysis. Moreover, the images displaying movement artifacts and those revealing cystic lesions or obstructive barriers in the area of interest were excluded. The study proposal was thoroughly reviewed and approved by the ethics committee of Mashhad University of Medical Sciences (IR.MUMS.DENTISTRY.REC.1400.085). All patients provided informed consent at the time of image acquisition, concerning the anonymous use of their data in future research. The study procedures were under the ethical standards mentioned in the Helsinki declaration.

The sample size was determined at n=90, based on the data obtained from Bertl et al,^18^ with an alpha significance level of 0.05 and a power of 80%. To enhance reliability, the sample size was later increased to n=100.

### IMAGE ACQUISITION AND ASSESSMENT

CBCT scans were acquired by the Planmeca Viso G7 apparatus (Planmeca, Helsinki, Finland), ensuring the Frankfort horizontal plane was parallel to the ground. The parameters employed were as follows: 90 Kv, 9 mA, a 90 × 90 mm field of view (FOV), and a 0.2-mm voxel size. The data were saved in DICOM (Digital Imaging and Communications in Medicine) format and subsequently imported into the Planmeca Romexis Viewer software (version 5.3.4) for further processing. 

The following protocol was adopted to quantify the root volume of maxillary canines and lateral incisors on both the impaction side and the normal eruption side. 

**Figure 1:**
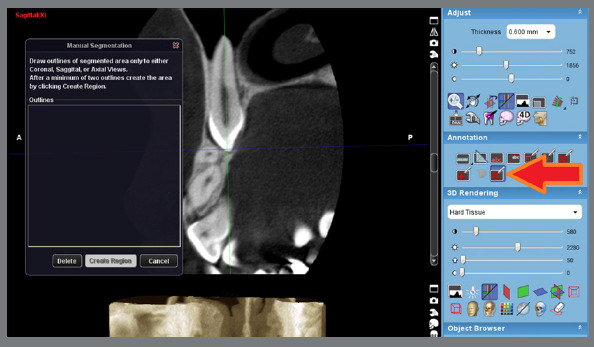
The longitudinal axis of the impacted canine is aligned with the sagittal plane. The manual segmentation tool in the Annotation part of the software can be used to divide the tooth into several layers.

**Figure 2:**
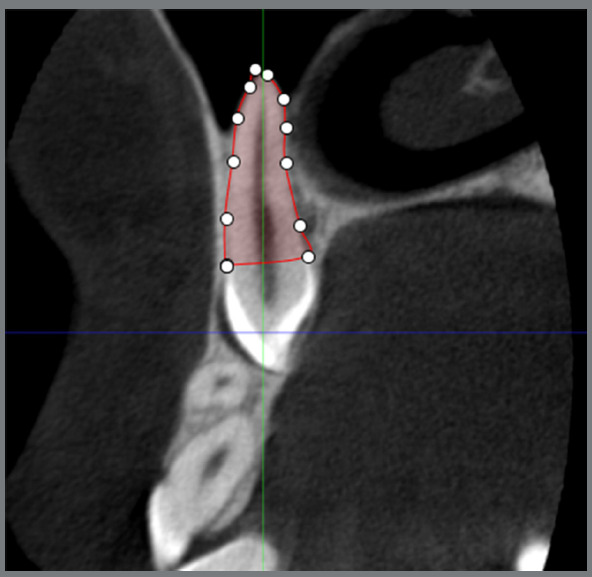
Marking the boundary of the impacted canine in the selected layer between the cementoenamel junction and apex.

Once the DICOM data was loaded into the Planmeca Romexis Viewer software, the targeted canine or lateral incisor was segmented and positioned at the center of the image (Fig 1). The image was reoriented to ensure the longitudinal axis of the tooth corresponded to the sagittal and coronal planes. The Manual Segmentation tool was employed from the “Annotation” section of the software, to create distinct layers (Fig 1). The layers were reconstructed between the cementoenamel junction (CEJ) and root apex at intervals of 600 µm (Fig 2). On each layer, the outer boundary of the tooth was demarcated by points, which were subsequently connected by lines, to form the tooth environment. The volume of each layer could be calculated by multiplying its surface area by its thickness of 600 µm.

**Figure 3:**
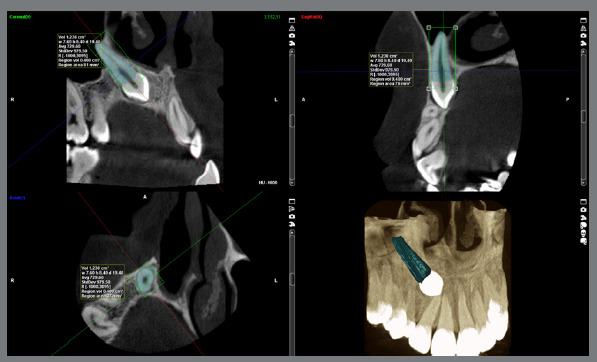
The root volume of the impacted canine is displayed in different views. In this case, the root volume ( Region vol ) was 0.400 cm^3^.

After delineating the environment of all selected layers, the “create region” command of the Planmeca Romexis Viewer was selected. This step enabled the software to calculate the root volume (cm³) and provide a visual representation of the tooth along with the corresponding data (Fig 3). In the particular case demonstrated in Figure 3, the root volume (Region vol) was calculated as 0.400 cm³.

The aforementioned procedure was repeated for all canines and lateral incisors, and the resulting data were recorded. The root volumes of canines and lateral incisors were classified as the impaction and the normal-eruption sides, and subsequently compared. To ensure consistency in the measurements, 10% of the samples were reevaluated after 7 days by the same examiner. The intra-observer reliability was then ascertained through the intra-class correlation coefficient. 

### STATISTICAL ANALYSIS

The Shapiro-Wilk test was employed to assess the normal distribution of the data. It indicated that data associated with palatal canine impaction conformed to normality (*p*>0.05), whereas the data concerning buccal impaction did not (*p*<0.05). Therefore, the differences in root volume measurements between the impaction and normal eruption sides were assessed by applying the paired *t*-test or Wilcoxon signed-rank test, where appropriate. The statistical analysis was conducted by SPSS 22.0 software (IBM Corp., Armonk, NY, USA) and the significance level was set at *p*<0.05.

## RESULTS

### PATIENT CHARACTERISTICS

Out of 100 subjects who had unilateral maxillary canine impaction, 62 (34 females, 28 males) demonstrated buccal canine impaction and 38 (15 females, 23 males) presented palatal impaction. The mean age of the participants was 20.8 ± 5.4 years, ranging from 16 to 42 years. The average intra-class correlation coefficient (ICC) value was found to be 0.959 (ranging between 0.902 and 0.986), implying that the volumetric measurements were highly reliable and reproducible.

Table 1 presents the mean and standard deviation (SD) of root volumes (mm^3^) for canines and lateral incisors in patients displaying buccal or palatal canine impaction. The mean root volume of buccally-impacted canines was found to be 548.8 mm^3^, while the corresponding volume for normally erupted canines was 509.7 mm^3^. The results of the Wilcoxon test revealed a significant difference in the root volume of canines between the two sides (*p*<0.001; Table 1). The mean root volume of lateral incisors was quantified as 373.1 mm^3^ on the side of the buccally-impacted canine, and 418.4 mm^3^ on the non-impacted side. The statistical analysis revealed that the root volume of lateral incisors was significantly smaller on the impacted side than on the non-impacted side (*p*<0.001; Table 1). 

**Table 1:** Comparison of root volumes (mm^3^) of canines and lateral incisors between impacted and non-impacted sides, in patients with buccal or palatal canine impaction.

		Impaction side		Non-Impaction side		P-value
		Mean	SD	Mean	SD	
Buccal impaction (n=62)	Lateral incisor	373.1	80.0	418.4	85.1	P<0.001*
	Canine	548.8	107.0	509.7	111.4	P<0.001*
Palatal impaction (n=38)	Lateral incisor	386.8	59.2	379.7	61.5	P=0.177**
	Canine	549.3	98.3	488.0	97.1	P<0.001**

SD = standard deviation; *Wilcoxon signed-rank test; ** Paired t-test.

Palatally-impacted canines exhibited a significantly larger root volume (549.3 mm^3^), compared to their contralateral, normally erupted (488.0 mm^3^) teeth (*p*<0.001; Table 1). Lateral incisors adjacent to palatally-impacted canines showed a mean root volume of 386.8 mm^3^, which was marginally exceeding that on the non-impacted side (379.7 mm^3^), although the difference was not significant (*p*=0.177; Table 1). 

## DISCUSSION

In the present study, the root volumes of maxillary canine and lateral teeth were compared between the impacted and non-impacted sides in CBCT images of 100 patients who presented unilateral maxillary canine impaction. The adoption of a split-mouth design allowed the comparison between the impaction and normal-eruption sides within the same patients, thus effectively eliminating inter-individual variability.^4^ The frequency of buccal canine impaction was roughly 1.5 times higher than that of palatal impaction in the present sample. In contrast, numerous studies argued that palatal impaction occurs more frequently than buccal impaction.^19^ ^-^ ^21^ Kim et al^1^ assumed that ethnic population could significantly influence the location of canine impaction, as the rate of buccal impaction in their sample of East Asians was three times higher than palatal impaction. 

The CBCT images and the Planmeca Romexis software were used in this study to create 3D reconstructed images for accurate measurement of root volumes of canines and lateral incisors. CBCT allows observation of complex anatomical structures in three dimensions, and yields detailed and valuable data in cases presenting severe eruption disturbances, with the added benefit of lower cost and radiation dose, when compared to medical CT.^20^ ^,^ ^22^ ^,^ ^23^ Through 3D reconstructed images, it is possible to localize maxillary impacted canines, determine the severity and extension of root resorption, and measure the crown and root sizes of canine and adjacent teeth.^13^ ^,^ ^24^ Dalessandri et al^25^ evaluated the reliability of a radiological index for assessing the treatment difficulty of impacted canines using three CBCT scanners (NewTom, Kodak, and Planmeca). Their results indicated no significant difference between the various scanners in terms of image evaluation, although the measurements conducted with the Planmeca Romexis software demonstrated superior reliability in the x- and y-axis values. 

The outcomes of this study revealed that the average root volume of impacted canine teeth was significantly larger than that of its normally erupted counterparts, irrespective of whether the tooth was impacted buccally or palatally. The greater root volume of canines on the impaction side may be due to developmental anomalies such as dilacerations or hypercementosis in the root region. While it remains undetermined whether the increased root volume is a cause or a consequence of canine impaction, it can be postulated that a discrepancy in the root size of maxillary canines between the two semi-arches, as depicted in CBCT images, could serve as an indicator of buccal or palatal canine impaction.

On the side of buccally-impacted canines, the roots of lateral incisors showed a significantly smaller volume than the normally erupted ones. Although the exact mechanisms underlying maxillary canine impaction are not well defined, it is clear that maxillary lateral incisors and canines share a significant interplay during the canine eruption. During the mixed dentition stage, the unerupted maxillary canine inclines mesially and leans over the root of the neighboring lateral incisor, which functions as a guide to direct canine eruption.^4^ ^,^ ^18^ It is worth noting that maxillary canines are the last teeth to erupt anterior to the first molars, suggesting that insufficient space in the dental arch would lead to buccal eruption or buccal impaction of canines. In contrast, palatally-impacted canines are usually observed in patients with excessive space in the dental arch. With these in mind, the diminished root size of the lateral incisor on the side of buccal canine impaction might arise from the root resorption at the apex induced by pressure from the canine’s crown. To better clarify this issue, future research should be performed using histopathological examinations to detect any signs of root resorption.

In this study, there was no significant difference in the root size of lateral incisors between the two sides, in cases presenting palatal canine impaction. Two theories have been put forth to elucidate the etiology of palatal canine impaction: the guidance theory and the genetic theory. Both theories focus on the critical role played by the lateral incisors in the normal eruption of maxillary canines.^5^ According to the guidance theory, the canine eruption is directed along the distal surface of the root of the maxillary lateral incisor. Consequently, the presence of interdental spacing or missing, peg-shaped, or small lateral incisors can compromise the guiding function of the lateral incisor, and the canine tooth would be allowed to migrate palatally.^4^ ^,^ ^5^ On the other hand, the genetic theory states that canine eruption is controlled by the same genes that regulate tooth size and the eruption of other teeth. As a result, palatal impaction is frequently observed in individuals who exhibit dental anomalies, such as maternal missing of lateral incisors or second premolars, or small lateral incisors.^4^ ^,^ ^5^ ^,^ ^26^ ^,^ ^27^

The findings from this investigation do not corroborate the theory that undersized lateral incisors contribute to the etiology of palatal canine impaction, because the root volume of maxillary lateral incisors was not significantly different between the sides of impaction and normal eruption. It should be noted that patients with missing lateral incisors or those exhibiting gross anomalies of lateral incisors were excluded from the sample. Therefore, other etiologies beyond the size of lateral incisors might contribute to the occurrence of palatal canine displacement. 

The outcomes of this study corroborate the results of Kim et al^3^, who examined the morphologic characteristics of maxillary dentition in 40 patients with unilateral maxillary canine impaction. They selected equal numbers (n=20) of buccal impaction and palatal impaction, and found that the width and volume of the canine crowns were significantly larger on the impaction side than the clinically normal eruption side. This finding led to the hypothesis that an increased crown size could be a potent factor influencing the impaired eruption of the maxillary canine.^3^ In another study, Kim et al^1^ reported an occurrence rate of 49.5% for adjacent root resorption in patients with impacted maxillary canines. They found that cases with buccally impacted canines demonstrated a higher degree of root resorption than those with palatally impacted canines. Liu et al^28^ reported that root volumes of canines were significantly bigger and those of lateral incisors were significantly smaller on the impacted side than on the non-impacted side. Ucar et al^29^ showed that the volume of the lateral incisor was significantly smaller on the impacted side, as compared to the normally erupted side, in patients with a unilateral impacted canine. However, they did not identify any statistical differences in the root resorption volume of lateral incisors, when comparing impacted canines on the labial or palatal sides. 

Contrary to the findings of this study, Dekel et al^30^ demonstrated that both buccally and palatally impacted canines exhibited shorter root lengths, compared to their contralateral counterparts. However, canine volumes were similar between the two sides in subjects with buccally impacted canines, and slightly smaller in instances of palatally displaced canines.^30^ Liuk et al^31^ exhibited that the mean length of lateral incisors was shorter by 2.1 mm, and the mean root width, in the buccolingual dimension, was smaller by 0.7 mm in patients displaying palatal canine displacement, compared to the control group. Leonardi et al^5^ identified shorter root lengths and smaller lateral incisor volumes on the side with palatally displaced canines, as compared to the contralateral side of the maxilla and the control group. Bertl et al^18^ highlighted a significant association between the apical root volume of lateral incisors and palatally-displaced canines (PDC). They postulated that a reduced apical root volume may not be the causal factor, but potentially a consequence of PDC.^18^

According to the outcomes of this study, it appears that the increased root volume of the canine on one side of the arch, compared to the contra-lateral side, could be representative of canine impaction. In instances where canines are assumed to be impacted, early interventions are of particular importance to improve the eruption path of canines and preclude negative sequelae in lateral incisors. These interventions involve immediate extraction of primary canines or performing arch expansion, to provide sufficient space for canine eruption and prevent adjacent root resorption. The present results do not support the assumption that a diminished root volume of the lateral incisor plays a significant role in the etiology of palatal canine impaction.

 One of the limitations of this study was its failure to explore the causal relationship among different variables. Furthermore, the crown sizes of canines and lateral incisors were not measured in this research. It is suggested that future studies examine the effect of factors such as the crown size, and the severity of canine impaction on the root volume of adjacent lateral incisors. Further research involving a larger sample size is needed to discern whether the variations in crown or root volume of canines between the two hemi-arches could contribute to the etiology of buccal or palatal canine impaction.

## CONCLUSIONS

According to the outcomes of this study:

1. The mean root volume of canines on the side of impaction was significantly larger than that on the side of normal eruption, whether the tooth was buccally or palatally-impacted. This pronounced discrepancy in canine root volume between the two sides, as viewed in CBCT images, could potentially serve as a marker to identify cases susceptible to an impaired canine eruption.
2. On the side of buccally-impacted canines, the roots of lateral incisors displayed a significantly smaller volume, when compared to those in the normal eruption side. The reduced root volume of the lateral incisor may be due to the root resorption at the apex induced by pressure exerted by the buccally-impacted canine’s crown.
3. There was no significant difference in the root size of lateral incisors between the two sides in cases presenting unilateral palatal canine impaction. This indicates that palatal canine displacement may also occur in subjects exhibiting normal lateral incisors and hence, factors other than the size of the lateral incisor could contribute to its manifestation.
